# Promising features of *Moringa oleifera* oil: recent updates and perspectives

**DOI:** 10.1186/s12944-016-0379-0

**Published:** 2016-12-08

**Authors:** Muhammad Nadeem, Muhammad Imran

**Affiliations:** 1 Department of Dairy Technology, University of Veterinary and Animal Sciences, Lahore, Pakistan; 2 Institute of Home and Food Sciences, Faculty of Science and Technology, Government College University, Faisalabad, Pakistan

**Keywords:** *Moringa oleifera* oil, Fatty acid profile, Antioxidant activity, Oxidative stability, Industrial applications

## Abstract

Lipids are the concentrated source of energy, fat soluble vitamins, essential fatty acids, carriers of flavours and many bio-active compounds with important role in maintaining physiological functions of biological body. *Moringa oleifera* is native to Himalaya and widely grown in many Asian and African countries with seed oil content range from 35–40%. *Moringa oleifera* oil (MOO) has light yellow colour with mild nutty flavour and fatty acids composition suggests that MOO is highly suitable for both edible and non-edible applications. MOO is extremely resistant to autoxidation which can be used as an antioxidant for the long term stabilization of commercial edible oils. Thermal stability of MOO is greater than soybean, sunflower, canola and cottonseed oils. High oleic contents of MOO are believed to have the capability of increasing beneficial HDL cholesterol and decreased the serum cholesterol and triglycerides. MOO applications have also been explored in cosmetics, folk medicines and skin care formulations. Overall, this review focuses on commercial production status, food applications, antioxidant characteristics, health benefits, thermal stability, fractionation, cholesterol contents, medicinal, nutraceutical action, toxicological evaluation, biodiesel production, personal care formulations and future perspectives of the MOO for the stake holders to process and utilize MOO as a new source of edible oil for industrial purpose.

## Background

Researchers are trying to find out the new and non-traditional sources of foods to feed the ever increasing human population. The diminishing resources of foods, particularly, edible oils and fats have necessitated to explore the potential of existing sources of edible oils. Fats and oils are the important part of diet in almost every civilization of the world. They are used in cooking, frying and baking etc. From nutritional point of view, fats and oils play an important role in body. They are the concentrated source of energy, fat soluble vitamins, essential fatty acids, carriers of flavours and many bio-active compounds, which are necessary for many physiological functions [1]. Dietary guidelines suggest that about 30% of the total caloric requirements should be fulfilled from fats and oils [2]. *Moringa oleifera* Lam (Drum Stick) is native to Himalaya and widely grown in many Asian and African countries [3]. Oil contents of the seeds range from 35–40% (Table 1) while the application of enzyme technology can further improve the recovery of oil from seeds using the orthodox oil extraction techniques [4]. In contrast to soybean, sunflower, canola, corn and cottonseed oil, *Moringa oleifera* oil (MOO) is extremely resistant to auto-oxidation [5, 6]. Fatty acids and triglyceride composition of oil suggests that MOO is highly suitable for both edible and non-edible applications (Table 2) [7]. Chemical characterization of oil revealed that free fatty acids, moisture content, refractive index, iodine value, peroxide value and colour unit (Lovibond Tintometer Scale) were 0.22 , 0.17%, 1.452, 65.7 cg/g, 0.24 (meqO_2_/kg), melting point 19.2 °C and Red 1.5 and 15 yellow (Table 3) [8]. MOO has light yellow colour, with pleasant nutty flavour. Electronic nose analysis also revealed the mild nutty flavour, resembling to peanuts, which was also evidenced by the overlapping of many chromatograms [9]. Due to pleasant nutty flavour, lower levels of peroxides, MOO is normally used without any pre-processing (Refining, Bleaching and Deodorization), which is mandatory for most of the commercial vegetable oils. As an example, soybean oil has dark red colour, beany flavour, canola oil has dark green colour with typical mustard smell and higher magnitudes of free fatty acids, phosphatides and other gross impurities, which must be removed to produce light coloured blend oils [10]. Oil processors treat free fatty acids and crude oil flavours as gross impurities and good quality edible oil should have lower concentration of these impurities. Substantial energy is required to convert crude oils into table oils. MOO has excellent oxidative and frying stability [11]. With 36.7% triolein was the major triglyceride, followed by palmito-diolein and stearodiolein (Table 4) [12]. Fatty acid composition of oil is almost similar to olive oil with oleic acid as the dominant fatty acid (75–77%) [6]. Studies of Rahman et al. [13] evidenced that concentration of oleic acid in high oleic version of MOO was greater than 80%, which was greater than olive oil. High oleic fraction of MOO can be regarded as functional food. Furthermore, studies have proved that diets rich in C_18:1_ has the potential to lower the serum cholesterol and minimize the risk of cardiovascular diseases. Oils rich in monounsaturated fatty acids are getting a great deal of attention of food industry for having healthful properties and better oxidative stability. MOO has a huge potential to become a commercial source of edible oil. However, promising features of MOO should be widespread. This work briefly describes the salient features of MOO as source of edible oil, for its probable use at commercial level. Proximate composition of *Moringa oleifera* seed revealed that the moisture content, crude protein, crude fat, ash content and nitrogen free extracts were 7.9, 38.3, 30.8, 4.5, 6.5 and 16.5%, respectively [7]. Oil content reported in this study was on the lower side while the studies of Anwar et al. [3] revealed that oil content of *Moringa oleifera* grown in temperate regions of Pakistan was 38–42%.

**Table 1:** Oil content of *Moringa oleifera* oil and some vegetable oils

Oil type	Oil content (%)	Reference
MOO	38–42	[22]
SFO	37–40	[52]
MKO	13–15	[53]
CSO	18–20	[54]
PKO	46–50	[15]
CHO	35–40	[55]
SBO	18–20	[56]
WSO	35–40	[54]
LSO	40–42	[55]

*MOO Moringa oleifera* Oil, *SFO* Sunflower Oil, *MKO* Mango Kernel Oil, *CSO* Cottonseed Oil, *PKO* Palm Kernel Oil, *CHO* Chia Seed Oil, *SBO* Soybean Oil, *WSO* Watermelon Seed Oil, *LSO* Lemon Seed Oil

**Table 2:** Fatty acid profile of *Moringa oleifera* oil and some vegetable oils

Fatty acid	MOO	SBO	CO	SFO	PO
C_12:0_	----	----	----	0.5	0.10
C_14:0_	----	0.1	----	0.2	1.24
C_16:0_	6.65	11	3.9	6.8	37.9
C_18:0_	2.82	4.0	1.9	4.7	4.11
C_18:1_	78.04	23.4	64.1	18.6	43.9
C_18:2_	4.16	53.2	18.7	68.2	13.4
C_18:3_	----	7.8	9.2	0.5	0.45
C_20:0_	2.46	0.3	0.6	0.2	0.38
C_22:0_	5.84	0.1	0.2	----	----
Reference	[21, 57]	[58]	[19]	[52]	[59]

*MOO Moringa oleifera* Oil, *SBO* Soybean Oil, *CO* Canola Oil, *SFO* Sunflower Oil, *PO* Palm Oil

**Table 3:** Comparison of chemical characteristics of *Moringa oleifera* oil with some vegetable oils

Parameter	MOO	SBO	CO	SFO	P. Olein	PO
FFA %	0.16	0.88	0.35	0.31	0.08	0.08
Moisture	0.17	0.18	0.15	0.19	0.11	0.14
*Colour	1.0 + 10	3.5 + 35	3.2 + 33	1.3 + 13	1.2 + 12	1.0 + 10
RI@40 °C	1.452	1.467	1.462	1.473	1.457	1.452
Sap. Value	192	189	195	192	191	194
USM	1.28	1.21	1.24	1.31	0.67	0.62
IV	65.7	133.7	114.5	121.8	56.2	53.1
PV	0.24	1.8	1.5	2.34	0.27	0.21
Reference	[4]	[56]	[52]	[60]	[15]	[15]

*MOO Moringa oleifera* Oil, *FFA* Free Fatty Acids, *Sap. Value* Saponification Value, *USM* Unsaponifiable Matter, *IV* Iodine Value, *PV* Peroxide Value, *SBO* Soybean Oil CO: Canola Oil, *SFO* Sunflower Oil, *P. Olein* Palm Olein, *PO* Palm Oil

*Lovibond Tintometer Scale (Red + Yellow) 1” Quartz Cell

**Table 4:** Triglyceride profile of *Moringa oleifera* oil and Virgin olive oil

Moringa oleifera oil	Virgin olive oil
OOL	PPO
OOLn	PPL
POL	PSO
OOO	POO
POO + SOL	POL
OOGa	PLL
SOO	SSO
OLA	SOO
OOA	OOO
PPO	SOL
PPL	OOL
PSO	OLL
POB	OOLn
Reference [9]	LLL
	OLLn
	Reference [61]

## Commercial production status

The situation of food insecurity is getting worst day by day, in future, feeding of ever increasing human population perhaps would be the most difficult task. On the other hand, resources of foods are drying. According to an estimate, about 2 billion humans will be added to the population of Asia and Africa [14]. In addition to other nutritional requirements, fats and oils must be consumed in sufficient concentration to fulfil the body’s requirements [15]. Natures has gifted about 500,000 edible oil producing plants, it is worth mentioning that only 12 are being utilized for commercial production and processing. In view of the existing situation of food insecurity in the third world countries, new sources of edible oils must be discovered. *Moringa oleifera* (Drum Stick) is extensively grown in tropical and subtropical regions of Asia and Africa [8]. Soybean, sunflower, cottonseeds are the leading oilseeds. Oil content of soybean and cottonseed are about 18–20%, with such moderate oil content. If soybean oil can become the leading source of edible oil, then how a plant (*Moringa oleifera*) with 40% good quality oil content cannot become the commercial source of edible oil. *Moringa oleifera* produces 3000 kg seed from 1 ha that can produce 1200 kg edible oil, as compared to soybean which produce 350–400 kg oil from 1 ha [6]. Due to lack of awareness, it is not commercially grown as an oilseed. India has adopted a wise strategy and started the commercial production of MOO, currently, 1.3 M. Ton of edible oil is annually extracted from the seeds of *Moringa oleifera*, with 380 KM^2^ area of production [16]. The cost of production of oil from *Moringa oleifera* is low as compared to other sources of edible oils, the unreferenced source revealed that cost of 1 kg seed is 0.15$. In addition to low cost and higher oil content, oil has better functional properties over soybean, sunflower, canola, corn oils, they need partial hydrogenation for improved functional properties, whereas, MOO does not require partial hydrogenation. It can also be converted into olein and stearin fractions, which only have better functional properties but can also serve as superior alternates of partially hydrogenated fats. Further it contains about 5–6% behenic acid, which act as crystallizing agent [4]. In subcontinent, crystallized vanaspati is preferred over pasty stuff andapplication of MOO in vanaspati can improve its graininess and crystallization behaviour. Oil production potential of *Moringa oleifera* was assessed in arid climate of Chaco South Africa, on average basis; it produced 481.25 Kg edible oil from one acre, desert conditions did not have significant effect on the seed production and oil content [17]. Commercial oilseeds require good quality soil with plenty of water, adequate fertilization and other expensive agronomic practices, whereas, Moringa tree can be grown in poor quality sandy, salt affected soils and it can resist long spells of drought with no effect on oil yield. The results of another investigation conducted in Argentina disclosed that *Moringa oleifera* trees produced 595 kg oil/acre in drought conditions [18].

## Food applications and antioxidant characteristics

Anwar et al. [19] blended sunflower, canola and soybean oils with MOO at various concentrations, blending modified the physico-chemical characteristics of these oils. Fatty acid compositions of blends were different from the substrate oils; storage studies revealed that blends of sunflower, soybean, canola oils with MOO, generated lower concentration of primary and secondary oxidation products during the long term storage, with improved induction period. These results evidenced that MOO can be used for the enhancement of oxidative stability of commercial edible oils. Functional fat was prepared by blending MOO and butter oil. MOO was used up to 50% concentration and blends were then interesterified by sodium methoxide. Melting point of blend containing 50% butter oil and 50% MOO was 35.5 °C, with considerable reduction in cholesterol. Results of storage and accelerated oxidation studies indicated that blends of butter oil with MOO were more resistant to oxidation [20]. The substantial antioxidant potential of MOO was utilized for the preservation of butter oil at ambient temperature; butter oil was blended with MOO from 2.5% to 10% concentration. Blending of butter oil with MOO enhanced C_18:1_, free radical scavenging activity of blend containing 10% MOO was 31.65% as compare to 5.22% in butter oil. The total phenolic contents of blend containing 7.5% MOO were 5.51 mgGAE/g. In accelerated oxidation conditions, blends containing MOO offered more resistance to auto-oxidation. The concentration of conjugated dienes and trienes were significantly lower in the blends as compared to butter oil [21]. Suitability of MOO in the formulation of vanaspati was studied and MOO was incorporated into palm olein up to 75% level and blends were transesterified with *Rhizopus miehei*. Blends were stored for 6 months and studied at 0, 90 and 180 days of storage. Melting point of blend containing 50% MOO was indifferent from market vanaspati with no harmful *trans* fatty acids. Concentration of C_18:1_ was 56.7%, when 50% MOO was added in the blend. MOO at all concentrations considerably improved the induction period, inhibited the oxidation of unsaturated fatty acids. A panel of trained judges was unable to find out any difference in the sensory characteristics of French fries prepared in vanaspati containing 50% MOO [22]. MOO has a melting point of 16–20 °C, with these melting characteristics it cannot be used as salad oil. Rahamn et al. [13] fractionated MOO into olein and stearin fractions. Olein fraction was regarded as high oleic acid fraction (HOF). C_18:1_ content of HOF was greater than olive oil; melting point of HOF was 1.2 °C with increased iodine value and lower cloud point. Oxidative stability of HOF in long term storage and accelerated oxidation was superior to sunflower, canola and soybean oils. MOO can replace the synthetic antioxidants for the long term preservation of butter oil at ambient temperature [21]. MOO oil was exposed to air to induce oxidation and oxidative stability of MOO was determined using peroxide value and specific extinctions at 232 nm. After long term storage, MOO showed superior oxidative stability with lower levels of peroxides and specific extinctions measured at 232 nm [23]. Anwar and Bhangar [8] studied the antioxidant potential of MOO; they recorded the existence of higher magnitudes of antioxidants in MOO. MOO contains substantial amount of behenic acid (5.8 to 6.2%), behenic acid is regarded as oil structuring and solidifying agent for potential application in margarine, shortenings without partial hydrogenation [24]. MOO was blended with palm olein, plam stearin and virgin coconut oil from 30 to 70% in the formulation of margarine, blending had major effects on solid fat index, melting behaviour, fatty acid, triglyceride profile. MOO improved the spreadability of margarine with zero *trans* isomers. MOO also enhanced the functional properties of palm stearin [25]. MOO oil contains zeatin, a potentially bioactive substance which is believed to have antioxidant and anti-inflammatory properties. It also contain beta-sitosterol, which blocks the biochemical events of cholesterol formation and possesses anti-inflammatory perspective. MOO is also a rich source of kaempferol, which improves the metabolism and cell function, 36 natural antioxidants are naturally present in MOO [26]. The data regarding the presence of sterols (Table 5), tocopherols (Table 6) and total phenolic contents (Table 7) have been presented, respectively.

**Table 5:** Sterols of *Moringa oleifera* oil and some vegetable oils mg/100 g

Sterol	MOO	SBO	CO	SFO
Campesterol	15.81	68	10.29	9.28
Stigma sterol	23.10	64	7.51	0.18
β-Sitosterol	45.58	183	58.01	50.28
∆^5^ - Avenasterol	8.46	5	1.26	1.11
∆^7^ – stigmasterol	Not Reported	5	9.72	0.11
∆^7^- Avenasterol	0.53	2	5.54	0.06
Reference	[23]	[62]	[63]	[63]

*MOO Moringa oleifera* Oil SBO: Soybean Oil CO: Canola Oil, *SFO* Sunflower Oil

**Table 6:** Tocopherol contents of *Moringa oleifera* oil and some vegetable oils

Oil type	Tocopherol	Concentration mg/kg	Reference
*Moringa oleifera* oil	α-Tocopherol γ-Tocopherol δ-Tocopherol	134.42 93.7 48.0	[3]
Virgin Olive Oil	α-Tocopherol γ-Tocopherol δ-Tocopherol	88.50 9.90 1.60	[23]
Watermelon Seed Oil	α-Tocopherol γ-Tocopherol δ-Tocopherol	127.49 ---- 55.36	[54]
Mango Kernel Oil	α-Tocopherol γ-Tocopherol δ-Tocopherol	205.44 ---- 34.81	[64]
Soybean Oil	α-Tocopherol γ-Tocopherol δ-Tocopherol	9.3 62.8 26.7	[65]
Sunflower Oil	α-Tocopherol γ-Tocopherol δ-Tocopherol	613 19 ----	[66]
Canola Oil	α-Tocopherol γ-Tocopherol δ-Tocopherol	226 202 3	[67]
Corn Oil	α-Tocopherol γ-Tocopherol δ-Tocopherol	134 412 39	[54]

**Table 7:** Total phenolic contents of *Moringa oleifera* oil, vegetable oils and extracts

Oil type	TPC % (GAE)	Reference
*Moringa oleifera* oil	7.1	[21]
Chia Oil (Olein	4.25	[68]
Chia Oil (Stearin)	2.57	[68]
Almond Peel	3.82	[22]
Sesame Cake	1.84	[22]
Chia Seed Extract	5.6	[54]
Sugarcane Juice	6.19	[55]
Date Fruit Extract	5.19	[69]
Tamarind seed	6.45	[70]

*TPC* Total Phenolic Contents, *GAE* Gallic Acid Equivalent

## Health benefits

The demand and production of monounsaturated oils is mounting across the globe and large number of health benefits and superior oxidative stability of monounsaturated oils has increased their application in large number of foods [11]. Oleic acid (*cis-*9-octadecanoic acid) possesses a cholesterol lowering properties [27]. Olive oil, canola oil, peanut and rapeseed oil are regarded as the rich source of oleic acid, which varies from 50 to 80% [11]. Dietary guidelines suggest that oleic acid should be the part of regular diet to minimize the risk of cardiovascular diseases [28]. Concentration of oleic acid has been increased in some oilseeds through genetic modifications, e.g. high oleic acid sunflower oil and canola oil [11]. Monounsaturated oils do not require partial hydrogenation for better shelf stability. Consumption of partially hydrogenated fats has been correlated with the development of cardiovascular diseases [29]. High oleic acid fraction of MOO has been developed by enzymatic transesterification and fraction [4]. With greater than 80% oleic acid, high oleic acid fraction may be appropriately regarded as power house of oleic acid [13]. Oleic acid also act as a precursor of omega-9 fatty acids, human body has the capability to convert C_18:1_ to omega-9 fatty acids.

## Thermal stability

A study was performed to investigate the frying stability of MOO, canola oil and sunflower oil. Frying test involved the frying of French fries for 6 days/ per, the total length of experiment was 6 days. Peroxide value, total polar compounds, conjugated dienes and trienes were used as indicators of oxidative deterioration. After 6 heating cycles (each cycle 6 h) peroxide value of canola oil and soybean oil was considerably higher than MOO. The rate of darkening and increase in viscosity in canola and soybean oils were substantially higher than MOO. The concentrations of total polar compounds were the lowest in MOO [4]. Anwar et al. [19] also reported that the concentration of polymers in MOO after repeated heating cycles were lower than soybean, canola and sunflower oils. Generation of polymers is a serious problem which not only reduces the nutritional quality of frying media but also has a bad impact on sensory attributes of fried goods. Melting point of *moringa oleifera* is similar to palm olein. Currently, potato chips frying industry is mainly concentrating on palm olein as frying stuff. MOO with great frying stability can be a used as an alternate of palm olein for the frying industry. Nadeem et al. [22] studied the sensory characteristics of French fries prepared in MOO and palm olein blends, sensorial perspectives of French fries were not different to commercially used fat. Oxidative stability of soybean, sunflower and canola oils was compared with high oleic acid fraction of MOO [13]. After 5 days in an oven (63 °C), peroxide values of sunflower, soybean and canola oils were greater than high oleic acid fraction of MOO [13]. The superior oxidative stability of MOO in accelerated oxidation conditions is also reported in literature (Table 8) [20, 21]. The higher oxidative stability of MOO during deep frying has been reported [5]. Oxidative stability of cold pressed MOO was compared with raw and groundnut oil, concentration of free fatty acids, peroxides and oxidation products was lower in MOO than raw and refined groundnut oil [30]. Frying performance of MOO was assessed in repeated frying process for 5 days at 175 °C and MOO revealed excellent frying stability, generation of lower amounts of free fatty acids, peroxides, extinction coefficients at 232 nm with minimum decline in flavour score [5].

**Table 8:** Induction period of *Moringa oleifera* oil and some vegetable oils

Oil Type	Induction period (Hours)	Reference
MOO	42.56 after degumming	[23]
MOO	72.56 crude oil	[23]
PO	10.4	[52]
SBO	4.27	[56]
CO	5.84	[19]
SFO	3.51	[19]
WSO	3.82	[54]
CHO	1.32	[71]
WSO	4.1	[54]

*MOO Moringa oleifera* Oil SFO: Sunflower Oil MKO: Mango Kernel Oil, *CSO* Cottonseed Oil, *CHO* Chia Seed Oil, *SBO* Soybean Oil, *WSO* Watermelon Seed Oil, *LSO* Lemon Seed Oil

## Cholesterol contents

Cholesterol is a steroid, waxy metabolite, belongs to the unsaponifiable matter of lipids. It is insoluble in the aqueous/watery phase of blood, transported in the body through specialized proteins to various parts of the body [31]. LDL carries cholesterol from liver to the peripheral tissues, LDL has been designated as bad cholesterol, high density lipoproteins (HDL) transport cholesterol back from the peripheral tissues to the liver and regarded as beneficial cholesterol [32]. The ratios of LDL to HDL, concentration of total cholesterol, LDL and triglycerides exceeding 5, 200, 150 and 150 and HDL lower than 35 mg/dL, respectively is associated with enhanced risk of cardiovascular disease. Intake of dietary cholesterol should not exceed 300 mg/day [33]. Dairy products e.g. cheese, cream, butter, butter oil etc. For example, 219, 110, 105 mg/100 g cholesterol is present in butter, cheese and ice cream, respectively [34]. MOO does not cholesterol, blending of butter oil with MOO significantly decreased the concentration of dietary cholesterol [20].

## Fractionation

Rahamn et al. [13] fractionated MOO into olein and stearin fractions through dry crystallization technique, fatty acid composition of olein fraction revealed the magnitude of oleic acid of oleic acid was greater than 80%. The effect of MOO on the crystallization behaviour of palm oil was investigated and MOO was incorporated into palm oil at 20% concentration. Fractionation was performed by solvent and dry crystallization techniques at 18 and 21 °C and the yield of olein fraction obtained via solvent fractionation was greater than dry crystallization. GC analysis of the liquid fractions revealed that the intensification of oleic acid and triolein. Remarkable differences were noted in the melting characteristics of olein and stearin fractions obtained by two different fractionation methods. MOO stearin has higher melting point and may be used as an alternated of chocolate fat/confectionary fat and bakery shortening, however, theses aspects needs intensive research work [35]. After the rearrangement of esters, melting point of MOO increased from 19.8 °C to 35.2 °C [20]. MOO was transesterified with lipases *meihei* followed by fractionation. Fatty acid composition, solid fat index of olein and stearin fraction was considerably different from the parent MOO, olein fraction remained clear at 2 °C, with no haziness, offering a great perspective for usage as salad/oil/cooking oil/mayonnaise oil etc. [4].

## Medicinal and nutraceutical action

MOO is primarily a high oleic version of essential/ edible oil, oleic acid possesses anti-inflammatory characteristics, prevents cardiovascular diseases and breast cancer (Table 9) [36]. Oil is a rich source of vitamin A, and E, with strong antibacterial properties and it confers softness and smoothness to dry and tuff skin. It also possesses antihypertensive, antifungal and antiepileptic characteristics [37]. It can be used in many types of soaps, for the treatment of rheumatism, gout and venomous bites [38]. Strong antioxidant activity of MOO oil can be utilized in the formulation of body cream with increased antioxidant activity, antibacterial activity, better inhibition of free radicals, smooth and soft skin [39]. MOO has been used in the preparation of folk medicines in many civilizations since pre-historic time. The therapeutic perspectives of MOO has been documented in literature and it has antiseptic and anti-inflammatory, anti-rash characteristics, cures insect bites, burns and cuts, fights black heads, acne, counterfeiting the bad effects of pollution of skin, maintain natural glow of the skin, scalp moisture, improves the strength of hairs, act as antidandruff and prevent split ends, unlike other vegetable oils. It is great source of vitamin C, which prevent scurvy and possesses many other health benefits and it has the capability of lowering hypertension and persuade good sleep, guards bones and calms the nervous system. MOO has been the part of skin ointments, skin moisturizing agent and other skin cosmetics since the Egyptian times [40, 41]. Efficacy of lovastatin and *Moringa oleifera* were compared and study trial was conducted on rabbits. Feed was supplemented with 6 mg/kg/day and 200 mg/kg/day. After the experimentation period of 120 days, it was observed that both *Moringa oleifera* and lovastatin reduced the serum cholesterol, phospholipids, triglycerides, VLDL and LDL cholesterol. Rabbits fed on *Moringa oleifera* supplemented diet showed less fat in liver, heart and aorta. Faecal analysis revealed that concentration of cholesterol in the faces of *Moringa* supplemented diet was higher than control group (which did not receive Moringa supplemented diet) [42].

**Table 9:** Therapeutic Perspectives of *Moringa oleifera* oil

Medical Disorder	Reference
Fungus induced infections	[5]
Pyodermia (Skin Disease)	[72]
Laxative	[49]
Improved prostate function, Scurvy,	[49]
Gout and bladder function	[21]
Antioxidant	

## Toxicological evaluation

The impact of MOO centred feed on growth performance, packed cell volume, haemoglobin, white blood cells, monocytes, lipid profile, urea, creatine were determined in albino rats. Three weeks old, 12 albino rats were randomly stratified in two groups, each group was comprised of 6 rats, the average weight ranged from 31.6 to 35 g. The group of rats fed on soybean oil was used as control, while, the second group was fed on MOO, experiment lasted for six weeks. After six weeks, blood samples of both the groups were tested for total cholesterol, high density lipoprotein, low density lipoprotein, triglyceride. Body weight, creatine, urea concentration and haematological parameters of both the groups were not significantly different. These results evidenced that MOO improved the growth performance and had a positive immune stimulatory impact on the growth of albino rats, with neutraceutical effect and no risk of cardiovascular disease [43].

## Biodiesel production

*Moringa* seeds produce 30–40% good quality oil, which is high in oleic acid. The quality of MOO is superior to sunflower oil and scientific evidences have shown that biodiesel prepared from MOO was superior to biodiesel made from other substrates [44]. The methyl esters of biodiesel prepared from MOO was 67, which is the highest for a biodiesel fuel. About 2000 l biodiesel can be produced from one hectare and production of biodiesel can be started after one year of the plantation, as tree bears fruit within one year of the cultivation [45]. The biodiesel derived from MOO has higher iodine value as compared to conventional diesel fuels, which indicates that MOO based biodiesel has better stability [46]. With higher octane number, it has higher ignition performance and cold filter plugging point, showing better ignition performance in winter as well. The recovery and quality of biodiesel from MOO is higher as compared to other crops with the recovery of top grade glycerine as by-product. MOO is a better sustainable source of biodiesel than other plants e.g. Jatropha, as *Moringa* is cultivated primarily for food [47, 48]. 30 days old Moringa plants were milled to mesh size 5, followed by the separation of liquid fraction from solid mass through filtration. The liquid was transferred to gas reactor. 1 kg volatile solids produced 580 L of gas, with 81% methane content [49].

## Personal care formulations

The major fatty acid in MOO is oleic acid, which is widely recommended in the preparation of pharmaceutical ointments. It has high cosmetic value, helps to remove dirt from the skin and is considered as superb cleansing agent. It has non-drying characteristics, can be easily blended with other essential oils; these properties make *moringa* oil as excellent massage oil. Oil can be used in the preparation of different types of soaps, cosmetic cream and lip balm. MOO based soaps has stable lather with better cleansing perspectives [50]. MOO has been the part of folk medicines since thousands of years; oil was used as perfume and skin lotion by the Egyptian, Roman and Greek communities [51]. Egyptians used MOO in the treatment of skin disorders, as smoothing, moisturizing and oiling agent for the treatment of dry skin and therapeutic massages. It has the capability of absorbing and retaining the flavouring compounds [40]. Currently MOO is widely used in the formulations of body creams, lotions, balms, scrubs and anti-hair fall formulations. In cosmetics, it is preferred over other oil as it does not leave greasy after feel [24].

## Conclusions

MOO can be sued in the formulation of vanaspati, margarine, bakery shortening, salad oil, cooking oil, frying of potato chips, frying fats in industry and restaurants while stearin fractions can be used as bakery fat.
